# Documentation of preventive screening interventions by general practitioners: a retrospective chart audit

**DOI:** 10.1186/1471-2296-11-21

**Published:** 2010-03-09

**Authors:** Emmanuel Ngwakongnwi, Brenda Hemmelgarn, Hude Quan

**Affiliations:** 1 Department of Community Health Sciences, University of Calgary, Calgary, Alberta, Canada; 2 Department of Medicine, University of Calgary, Calgary, Alberta, Canada

## Abstract

**Background:**

Screening and early diagnosis has been shown to reduce the morbidity and mortality associated with certain conditions such as cervical cancer. The role of general practitioners in promoting primary prevention of diseases is particularly important given that they have frequent contact with a large proportion of the population. This study assessed the extent to which general practitioners documented recommended preventive screening interventions among eligible patients.

**Methods:**

We used a retrospective chart audit to assess patient visits to primary care clinics in Calgary, Canada from 2002-2004. We included fee for service physicians who practiced ≥ 2 days per week at their current location and excluded those whose primary practice was at walk-in clinics, community health centers, hospitals or emergency rooms. We included charts of patients who during the study period were age 35 years or older and had at least 2 visits to a clinic. We randomly selected and reviewed charts (N = 600) from 12 primary care clinics and abstracted information on 6 conditions recommended for preventive screening. Opportunities for preventive screening were determined based on recommendations of the Canadian Task Force on Preventive Health Care, the American College of Physicians, and the Canadian Cancer Society. Our main outcome measures included cancer screening (mammography and pap smears), immunization (influenza and pneumococcal), and risk factor assessment (cholesterol measurement and smoking cessation consultation).

**Results:**

Patient visits to GP clinics present opportunities for preventive screening. However, we found that documentation of interventions was low, ranging from 40.3% (cholesterol measurement) to 0.9% (pneumococcal vaccination) within 1 year, and from 67.4% to 1.8% within the prior 3 years.

**Conclusions:**

Documentation of preventive screening interventions by general practitioners was relatively low compared to the number of patients eligible for preventive screening. Some physicians opt to screen for PSA and DRE which is not recommended by the Canadian Task Force on Preventive HealthCare.

## Background

Screening and early diagnosis has been shown to reduce the morbidity and mortality associated with certain conditions such as cervical cancer, where screening combined with treatment can reduce cervical cancer mortality by about 70% [1,2]. Measuring the extent to which preventive care is actually undertaken in general practice settings is difficult. Some studies have used recommended screening recorded in medical or administrative records of patients to ascertain completed screening [3,4]. In cancer screening for example, receiving a recommendation from a healthcare provider is an important determinant of adherence to cancer screening guidelines [5-7]. However, missed opportunities for preventive screening have been documented in clinical encounters [3,5,7].

General practitioners (GPs) have frequent contact with a large proportion of the population and could play a particularly important role in promoting primary prevention by recommending screening tests. In the fee-for-services healthcare system, GPs in Canada are paid by volume of patients, without consideration of quality of care. During physician-patient encounters, time becomes crucial in assessing patient needs and providing treatment. With aging population, chronic diseases become prevalent and consume the most of GPs' available time [8]. Thus, time constrains limit the ability of physicians to comply with preventive services recommendations [9]. Given the constrains pose by time, and the relatively low rate of physician adoption of some clinical guidelines in general [10-12], the extent to which GPs document recommended screening is not well understood. The purpose of this study was to assess the extent to which GPs document recommended preventive screening interventions among eligible patients. Such knowledge can help developers of guidelines and health decision makers to design effective ways to improve preventive screening.

## Methods

### Design and Setting

This was a retrospective case audit of patients' medical records at GP clinics in Calgary. We choose Calgary urban over rural because of the heterogeneity in GP practice and convenience of data collection. Using lists of GPs obtained from the Alberta provincial licensing physician directories in Canada; we randomly selected and approached 20 practices with verified phone numbers, in Calgary, to determine their eligibility and to invite them (offering incentives) to participate in the study.

We included fee for service PGs who practiced > 2 days per week at their current location from 2002-2004 and excluded PGs whose primary practice was at walk-in clinics, community health centers, hospitals or emergency rooms. These were excluded because most of their patients do not see them regularly. Given that recommended annual medical examinations are routinely conducted by GPs, this exclusion criteria was aimed to include physicians with consistent opportunities to recommend screening for certain conditions. Ethics approval was obtained from the University of Calgary Conjoint Health Research Ethics Board. Physicians who agreed to participate completed a consent form.

### Chart data collection and determining patient eligibility

We randomly selected 50 charts from each of 12 eligible primary care clinics in the city of Calgary for patients who: 1)were age 35 years or older, 2)had at least 2 visits to a GP clinic between 2002-2004. This gave a sample of 600 patients (i.e. 550 paper charts from 11 clinics and 50 electronic medical records from 1 clinic). A sample size of 400 or more would have sufficient power to determine the frequency of preventive care within 5% of the true value with a precision of 95%.

Two nurses underwent training in the data abstraction process by reviewing selected charts individually, and subsequently together to reach consensus were discrepancies existed. We developed a data abstraction form (table 1) to record information under three sections (medical history, blood pressure reading, and GP recommendations). Using the data abstraction form, reviewers independently abstracted information from charts and electronic medical records, including demographic data, and recommendations for cancer screening (mammography and pap smears), immunization (influenza and pneumococcal), and risk factor assessment (cholesterol measurement and smoking cessation) in the prior three years (i.e. 2002-2004).

**Table 1:** Data extraction form: GP recommendations section

GP Recommendation	Date Recommended		Date Last done (D/M/Y)	
27) Mammogram	Yes	No	Yes	No
28) Pap Smear	Yes	No	Yes	No
29) PSA	Yes	No	Yes	No
30) Rectal Exam	Yes	No	Yes	No
31) Cholesterol	Yes	No	Yes	No
32) Influenza Vaccine	Yes	No	Yes	No
33) Pneumococcal Vaccine	Yes	No	Yes	No
34) Smoker	No			
N/R	Yes	Cessation discussed	Yes	No
35) Has had hysterectomy	N/A	No	Yes	

Eligible patients for each of 6 conditions were determined based on the recommendations of the Canadian Task Force on the periodic Health Examination (now the Canadian Task Force on Preventive Health Care-CTFPHC) [13], the American College of Physicians [14], and the Canadian Cancer Society [15]. Given that the CTFPHC recommends against performing the PSA and DRE for asymptomatic men, we extracted data to assess the 'likely inappropriate' use of the PSA and DRE. In this study, we used only data from the 'GP recommendation' section of the abstraction form. An intervention was considered documented if it was recorded in the medical record the actual date that the intervention was completed, or the date it was recommended by the GP. With proper documentation, and the absence of missed opportunities for preventive screening, we would expect documented interventions to properly reflect screening rates. However, it is worth noting that recording rates of recorded screening over a 1 and 3 year period is some what blunt given the different frequencies for which screening is recommended (some only 3 yearly-table 2).

**Table 2:** Number of opportunities for intervention identified and documented among patients presenting at GP clinics (N = 600).

Intervention	Criteria for determining patient eligibility*	Patients eligible for intervention in 2004	Documented need for intervention in 1 year (2004)	Documented need for intervention in prior 3 years (2002--2004)
Mammography for breast cancer	Biannual screening --for women aged 50 -- 69 years	142	40(28.2%)	61(43.0%)
Papanicolaou smear for cervical cancer	Screen sexually active women at least every 3 years^†^	399	59(14.8%)	99(24.8%)
Cholesterol measurement	Patients over 30 years of age, who either smoke, have hypertension, or diabetes mellitus.	233	94(40.3%)	157(67.4%)
Influenza vaccination	Patients over 65 years of age	114	17(14.9%)	35(30.7%)
Pneumococcal vaccination	Vaccination every 6 years of selected high risk populations Patients over 65 years of age	114	1(0.9%)	2(1.8%)
Smoking cessation	Advise smokers to quit or refer to smoking cessation counseling programs	99	3(3.0%)	19(9.2%)
^‡^Documented Inappropriate screening for PSA and DRE				
Prostate specific antigen test for prostate cancer (PSA)	Men over 50 years of age (not required)	106	8(7.5%)	35(33.0%)
Digital rectal examination (DRE) for prostate cancer	Annual examination for men over 50 years of age (not required)	106	20(18.9%)	47(44.3%)

*Criteria used to identify opportunities for preventive intervention based on recommendations of the Canadian Task Force on the periodic Health Examination [6], the American College of Physicians [7] and the Canadian Cancer Society [8].

^†^Sexual activity not defined -- all women considered

^‡^The Canadian Task Force recommends that PSA and DRE not be performed

The study sample was evaluated to determine if the frequency of preventive care was influenced by clustering at the level of GP clinic. The intra-cluster correlation coefficient (ICC = 0.1) was calculated from an ANOVA as follows:

Where,

MSB is the mean square between practices

MSW is the mean square within practices

k is the average number in each practice (50)

Descriptive statistics were used for demographic variables and to compute the proportion of interventions documented for each condition, and documented inappropriate screening for PSA and DRE.

## Results

We randomly selected and reviewed 600 charts (550 paper and 50 electronic records) from 12 GP clinics to determine patient eligibility for preventive screening and to assess documentation of preventive screening interventions for all 6 conditions considered. Overall, 67% of the 600 charts belonged to female patients. The mean age of patients was 54 years, ranging from 31.6 to 98.2 years. Among 12 clinics sampled, and for all eligible patients, we found that actual preventive screens documented ranged from 40.3% (cholesterol measurement) to 0.9% (pneumococcal vaccination) within 1 year, and from 67.4% to 1.8% within the prior 3 years. For the prior 3 years, and for all 6 conditions, this represents on average 29.5% of preventive screening opportunities that were documented (supposedly addressed by GPs) compared to 70.5% on average that remained undocumented (supposedly unaddressed or missed opportunities). Likewise, documented 'inappropriate screening' ranged from 7.5% within 1 year to 33.0% within the prior 3 years for PSA and from 18.9% to 44.3% for DRE (table 2).

## Discussion

We found that patients visiting GP clinics present several opportunities for preventive screening; however, actual documentation of the interventions remained low (29.5% on average in 3 years). Amongst eligible patients, we found much variation in rates of implementation between conditions (For example, 40% screening for cholesterol compared to 0.9% for pneumocaccal vaccination in one year). This apparent disparity in implementation may be attributed to the fact that the available information on patient medical history, blood pressure readings, most likely influenced GP decisions on recommending screening for cholesterol over other conditions. However, this was not independently verified in our study. Considering that 67% of the charts reviewed for this study belonged to females, one would have expected that GPs have opportunities to recommend screening for Pap smears given that most females were eligible. However, the relatively low rates observed suggest certain factors including doctor gender, may have had an influence on the doctor offering the intervention, or on uptake as women tend to select women for these tests. Due to the small sample of participating clinics (12), we did not analyze GP gender matching factors; nor did we record the gender of GPs.

The high number of undocumented interventions is of concern and raises questions about implementation of preventive screening recommendations by GPs. Our results are similar to that of another study [11], where compliance with recommendations remains low. A study of Ontario physicians found that only a low proportion of physicians adhere to breast cancer screening recommendations, and the proportion was even lower among physicians in cities [16]. In Calgary, missed opportunities for preventive screening have also been demonstrated in general internal medicine. In this setting the digital rectal examination for prostate cancer and cholesterol measurement were addressed most commonly, while papanicolaou smear for cervical cancer and pneumococcal vaccination were addressed less commonly [3].

The reasons for low rates of preventive screening practices are likely multi-factorial. In the case of PSA and DRE for example, low rates of documented screening are understandable given that both procedures are not supposed to be performed; thus the documented rates for PSA and DRE observed in our study are 'inappropriate' according to CTFPHC. We reviewed documented need for pneumoccal vaccination in prior 3 years (1.8%) which is 3 years short of the guideline recommended vaccination every 6 years of selected high risk population's of patients over 65 years of age. Even if this rate were doubled to 4% as expected over 6 years, this rate would still be low given the proportion of patients eligible for vaccination.

Several studies [16-18] have documented numerous barriers (patient, physician, social, and practice characteristics) to adoption of preventive guidelines. Strategies to promote preventive health by GPs include information transfer, learning through social influence, feedback, reminders, organizational reminders, financial incentives, and regulatory interventions [19-23]. Multifaceted interventions (combination of information transfer and learning through social change or management support) have been shown to be more effective than single interventions (using information transfer) [20,22,23]. Studies have shown that alternative payment approaches with bonuses for targeted preventive care [24] or incentives that elevate the revenue of GPs [25] may lead to an increase in preventive care interventions to eligible patients.

This study was limited by methodological issues inherent in undertaking a retrospective chart audit. GPs may recommend specific interventions but fail to document them. This was missed in our study. As a consequence, there is a possible underestimation of proportions due to the fact that advice on preventive activities or the activities themselves may have been provided but not documented in the charts. This is likely the case for topics such as smoking which do not require a specific intervention besides advice for smoking cessation. Another important limitation is that we did not explore reasons for the low rates of interventions documented. A likely reason is that GPs may have known (through patient GP conversations) that the patient had already had the preventive service provided in another service therefore not offered it. In addition, physician gender has been shown to improve screening for certain conditions including pap testing [26], however we did not perform GP-gender analysis in this study. Finally, we studied 12 primary care clinics in Calgary urban and were uncertain about the situation in rural and other geographic areas. Thus our findings may not be generalized beyond Calgary.

## Conclusion

Amongst eligible patients, documentation of preventive screening interventions was generally low. This has negative implications on preventive care, and might increase the burden of preventable chronic conditions and higher health care costs on the population and public in general. Improving preventive screening by GPs would require a much better investigation of the reasons for the low rates of recommending screening for eligible patients, GP practice setting, knowledge of guidelines, perceptions on guidelines, available incentives, among others. Our findings indicate the extent to which GPs document preventive screening interventions and do not accurately reflect adherence to recommended guidelines, thus, the results should be interpreted with caution.

## Competing interests

The authors declare that they have no competing interests.

## Authors' contributions

EN carried out the data analysis and prepared the initial draft. BN revised the draft for intellectual content and style. HQ contributed to data acquisition, study design, and analytical techniques. Both BH and HQ reviewed several drafts and made suggestions for revision. All authors read and approved the final manuscript.

## Pre-publication history

The pre-publication history for this paper can be accessed here:

http://www.biomedcentral.com/1471-2296/11/21/prepub

## References

[B1] Miller AB Anderson G Brisson J Laidlaw J Le Pitre N Malcolmson P Report of a National Workshop on Screening for Cancer of the Cervix CMAJ 1991 145 10 1301 1325 1933712 PMC1335946

[B2] National Cancer Institute of Canada Canadian Cancer Statistics 2004 report

[B3] Brull R Ghali WA Quan H Missed opportunities for prevention in general internal medicine CMAJ 1999 160 1137 40 10234343 PMC1230265

[B4] Kinsinger L Harris R Qaqish B Strecher V Kaluzny A Using an office system intervention to increase breast cancer screening J Gen Intern Med 1998 13 507 14 10.1046/j.1525-1497.1998.00160.x 9734786 PMC1497003

[B5] Zapka JG Lemon SC Interventions for patients, providers, and healthcare organizations Cancer 2004 101 1165 87 10.1002/cncr.20504 15329892

[B6] Finney Rutten LJ Nelson DE Meissner HI Examination of population wide trends in barriers to cancer screening from a diffusion of innovation perspectives (1987-2000) Prev Med 2004 38 258 68 10.1016/j.ypmed.2003.10.011 14766107

[B7] Institute of Medicine Hewitt M, Simone J Ensuring quality cancer care 1999 Washington, DC: National Academy Press

[B8] Ostbye T Yarnall KSH Krause KM Pollak KI Gradison M Michener JL Is there time for management of patients with chronic diseases in primary care? Ann Fam Med 2005 3 209 214 10.1370/afm.310 15928223 PMC1466884

[B9] Yarnall KSH Pollak KI Ostbye T Krause KM Michener JL Primary care: Is there enough time for prevention? Am J Public Health 2003 93 635 641 10.2105/AJPH.93.4.635 12660210 PMC1447803

[B10] Cabana DM Rand SC Powe RN Wu AW Wilson HM Paul-Andre CA Rubin RR Why Don't Physicians Follow Clinical Practice Guidelines?: A Framework for Improvement JAMA 1999 282 15 1458 1465 10.1001/jama.282.15.1458 10535437

[B11] Weingarten S stone E Hayward R Tunis S Pelter M Pharm D Huang H Kristopatis The adoption of preventive care practice guidelines by primary care physicians: do actions match intentions? J Gen intern Med 1995 10 138 144 10.1007/BF02599668 7769470

[B12] Grilli R Apolone G Marsoni S Nicolucci A Zola P Liberati A The impact of patient management guidelines on the care of breast, colorectal and ovarian cancer patients in Italy Med care 1991 29 50 63 10.1097/00005650-199101000-00005 1986177

[B13] Canadian Task Force on the Periodic Health examination The Canadian guide to clinical preventive health care; 1994. CMAJ 1999 160 Ottawa: Health Canada 1137 40 Cat no H21-117/19994E

[B14] Prostate cancer Can it be prevented? 2007 Canadian Cancer Society

[B15] Hayward RSA Steinberg EP Ford DE Roizen MF Roach KW Preventive care guidelines Ann Intern Med 1991 114 758 83 2012359 10.7326/0003-4819-114-9-758

[B16] Abdel-Malek N Chiarelli AM Sloan M Stewart DE Mai V Howlett RI Influence of physician and patient characteristics on adherence to breast cancer screening recommendations European Journal of Cancer Prevention 2008 17 1 48 53 10.1097/CEJ.0b013e32809b4cef 18090910

[B17] Balk SJ Landesman LY Spellmann M Centers for disease Control and Prevention lead guidelines:do pediatricians know them? J Pediatr 1997 131 2 325 7 10.1016/S0022-3476(97)70177-X 9290627

[B18] Olesen F Lauritzen T Do general practitioners want guidelines? Attitudes toward a county-based and a national college-based approach J Prim Health care 1997 15 141 145 10.3109/02813439709018504 9323781

[B19] Hulscher EJM Wensing M Grol PR Weijden T Weel C Interventions to Improve Delivery of Preventive Services in Primary Care Am J Public Health 1999 89 737 746 10.2105/AJPH.89.5.737 10224987 PMC1508735

[B20] Wensing M Weijden T Grol PR Implementing guidelines and Innovations in general practice: which interventions are effective? British Journal of General practice 1998 48 991 997 9624774 PMC1409988

[B21] Litzelman KD Dittus SR Miller EM Tierney MW Requiring physicians to respond to computerized reminders improves their compliance with preventive care protocols Journal of general internal Medicine 1993 8 6 311 317 10.1007/BF02600144 8320575

[B22] Baskerville NB Hogg W Lemelin J Process evaluation of a tailored multifaceted approach to changing family physician practice patterns improving preventive care J fam pract 2001 50 3 242 249 11252222

[B23] Hogg W Lemelin J moroz I Soto E Russell G Improving prevention in primary care: Evaluating the sustainability of outreach facilitation Can Fam Physician 2008 54 5 712 720 18474705 PMC2377224

[B24] Rimer BK Trock B Balshem A Engstrom PF Rosan J Lerman C Breast screening practices among primary physicians: reality and potential J AM Board Fam Pract 1990 3 26 34 2305638

[B25] Martin MC Hogg EW How family physicians are funded in Canada Medical Journal of Australia 2004 181 2 111 112 15257654 10.5694/j.1326-5377.2004.tb06190.x

[B26] Grewal S Bottorff JL Balneaves LG A Pap test screening clinic in a South Asian community of Vancouver, British Columbia: challenges to maintaining utilization Public Health Nursing 2004 21 412 418 10.1111/j.0737-1209.2004.21504.x 15363021

